# Inhibition of Hepatitis B Virus (HBV) replication and antigen expression by *Brucea javanica* (L.) Merr. oil emulsion

**DOI:** 10.3389/fcimb.2023.1193775

**Published:** 2023-07-25

**Authors:** Bo Qin, Shu Shen, Juan Lai, Wei Yang, Lili Feng, Jiefeng Ding

**Affiliations:** ^1^ Clinical Laboratory, Shaoxing Maternity and Child Health Care Hospital, Shaoxing, China; ^2^ Obstetrics and Gynecology Hospital of Shaoxing University, Shaoxing, China; ^3^ Department of Gynecology, Shaoxing Maternity and Child Health Care Hospital, Shaoxing, China; ^4^ GeneMind Biosciences Company Limited, Shenzhen, China; ^5^ Department of Anesthesiology, Shaoxing Maternity and Child Health Care Hospital, Shaoxing, China

**Keywords:** *Brucea javanica* (L.) Merr. (BJ), Bruceine B, Hepatitis B virus (HBV), hepatocellular carcinoma (HCC), interleukin-6 (IL-6), nucleotide analogs (NAs)

## Abstract

**Introduction:**

The seeds of Brucea javanica (L.) Merr. (BJ) have been traditionally used to treat various types of cancers for many years in China. In this study, we systematically investigated a BJ oil emulsion (BJOE) produced from BJ seeds with the purpose of evaluating its antiviral effect against hepatitis B virus (HBV).

**Methods:**

HepG2.215 (a wild-type HBV cell line), HepG2, and Huh7, transfected with wildtype (WT) or lamivudine-resistance mutant (LMV-MT) HBV replicon plasmids, were treated with different doses of BJOE and then used for pharmacodynamic evaluation. Cell viability was determined using CCK8 assay. The levels of HBsAg/HBeAg in cell cultured supernatant, HBcAg in cell lysis solution, and HBV DNA in both were evaluated.

**Results:**

BJOE at ≤5 mg/ml was nontoxic to carcinoma cell lines, but could significantly inhibit WT/LMV-MT HBV replication and HBs/e/c antigen expression in a dose-dependent manner by upregulating interleukin-6 (IL-6), demonstrating that it possesses moderate anti-HBV activity. As one of the major components of BJOE, bruceine B was found to play a dominant role in IL-6 induction and HBV inhibition.

**Discussion:**

Our results demonstrated that BJOE suppressed HBV replication by stimulating IL-6, indicating that it has promising clinical therapeutic potential for both WT and LMV-MT HBV.

## 1 Introduction

Hepatitis B virus (HBV) is a major global health problem and is ranked among the top 10 infectious diseases causing death (Ma et al., 2020). Patients with chronic hepatitis B (CHB) infection have a significantly higher risk of developing cirrhosis, liver failure, and hepatocellular carcinoma (HCC) (Qin et al., 2020). HBV-infected patients have an approximately 100-fold-increased risk for HCC (Qiao and Luo, 2019). According to the most recent global epidemiological statistics, over 2 billion people have serological evidence of previous or current HBV infection, and an estimated 257 million people are living with HBV infection (Pollicino and Caminiti, 2021). Due to the availability of effective vaccines against HBV, its threat as an infectious disease has gradually receded. However, approximately 882,000 people die each year globally from liver disease caused by HBV infection, and 300,000 of them reside in China (Xu et al., 2021). As a member of hepadnaviridae, HBV possesses reverse transcriptase (RT), similar to human immunodeficiency virus (HIV) (Yasutake et al., 2018). Due to error-prone RT, HBV exists in the form of quasispecies (Park et al., 2003). There are two main classes of anti-HBV reagents: alpha interferon (IFN-α) and nucleotide analogs (NAs) (Qin et al., 2013). IFN-α exerts anti-HBV activity by direct antiviral, immunomodulatory, and anti-proliferative effects (Lim et al., 2018). NAs, consisting of lamivudine (LMV), adefovir dipivoxil (ADV), entecavir (ETV), telbivudine (LdT), and tenofovir (TDF), target HBV-RT and effectively control HBV replication by mimicking physiological nucleosides (Pei et al., 2017). NAs incorporate into newly synthesized DNA chains and inhibit synthesis by terminating DNA replication (Yasutake et al., 2018). However, their anti-HBV efficacy could be significantly diminished by resistance mutations (Zoulim and Locarnini, 2009) and an increase in viral load, serum alanine aminotransferase (ALT), and aspartate aminotransferase (AST), ultimately leading to hepatopathy progression (Qin et al., 2017). As a result, antiviral therapy of HBV remains a major challenge.

Plant-derived substances produce a variety of metabolites with novel structures and interesting biological activities. *Brucea javanica* (L.) Merr. (BJ) is a plant belonging to the Simaroubaceae family, which is widely distributed in south China (Ye et al., 2015). According to the record of Compendium of Materia Medica written by Shizhen Li in the Ming Dynasty (1368–1644 AD), BJ seeds were bitter, cold, and toxic in nature. They were often used to treat various diseases, including cancers, dysenteric disorders, malaria, and gastric ulcer (Zhang et al., 2018). In addition, BJ also showed dramatic antiphytoviral activity. Bruceine-D extracted from BJ seeds exhibited significant inhibitory activity against tobacco mosaic virus infection and replication (Tan et al., 2015). Quassinoids isolated from BJ seeds could inhibit pepper mottle virus in pepper (Ryu et al., 2017). In the present study, BJ oil emulsion (BJOE) was found to inhibit HBV *in vitro* by upregulating interleukin-6 (IL-6), not only the expression of s and e antigen, but also HBV replication. IL-6 was reported to be closely associated with the HBV life cycle. IL-6 mediated control of HBV infection at the transcriptional level through activating the MAPK ERK and JNK, and thus down-regulated the expression of HBV key transcription factors, including HNF4α and HNF1α (Hösel et al., 2009). HBV transcription can be suppressed by IL-6 by targeting the epigenetic control of cccDNA (Palumbo et al., 2015). IL-6 can effectively inhibit HBV replication via the reduction of viral transcripts/core proteins and of HBV genome-containing nucleocapsids formation (Kuo et al., 2009). More importantly, IL-6 inhibits HBV entry through down regulating the human liver bile acid transporter Na^+^/taurocholate cotransporting polypeptide (NTCP), which had been identified as an HBV specific receptor (Bouezzedine et al., 2015). Bruceine B was shown to be responsible for the IL-6 upregulation and anti-HBV activity. Benefited by different antiviral mechanisms from NAs, BJOE showed same antiviral effect on both wildtype (WT) and lamivudine-resistance mutant (LMV-MT) HBV. Taken together, BJOE has promising therapeutic potential for CHB patients, either alone or in combination with NAs. Furthermore, BJOE may play a dual role for cancer patients combined with HBV infection.

## 2 Materials and methods

### 2.1 Cell culture and cell viability

Cells were maintained in Dulbecco’s modified Eagle medium (DMEM) supplemented with 10% fetal bovine serum, 100 U/ml penicillin, and 100 μg/ml streptomycin. In addition, to maintain the stably transfected dimeric HBV genome, HepG2.2.15 cells, stably carrying two dimers of the HBV genomic DNA (3’-3’ with respect to one another), were cultured in the presence of 500 μg/ml of G418 (Sells et al., 1987). HuH7 and HepG2 cell lines were obtained from American Type Culture Collection (ATCC, Manassas, VA, USA). HepG2.2.15 cells were provided by the Department of Infectious Diseases, Nanfang Hospital, Southern Medical University (Guzhou, People’s Republic of China).

Cell proliferation was measured with a CCK-8 kit (Dojindo, Gaithersburg, MD, USA). Cells were seeded into 96-well plates at 2 × 10^4^ cells per well. After the cells adhered to the well, PTD-p37 or PTD-p40 was added. At the indicated time, the CCK-8 agent was added to the wells and incubated at 37°C for 2 h. The absorbance at 450 nm was assessed using a microplate reader (BioTek Instruments, Winooski, VT, USA). Each experiment was performed in triplicate and independently repeated three times.

### 2.2 Reagents

BJ oil (0.9 g/ml) (MPN: Z19993152) was provided by Jiangsu Jiuxu Pharmaceutical Co., Ltd. (Jiangsu, China). Briefly, 0.5 ml BJ oil was mixed with 0.12 ml Tween-80 and 0.125 ml glycerinum, and sterilized distilled water was added to make up a total volume of 5 ml. The mixture was filtered with a 0.22-μm filter (Merck Millipore Ltd, Carrigtwohill, Cork, Ireland) to obtain 10% 90 mg/ml BJOE. Subsequently, the mixture was diluted with DMEM into obtain a concentration of 50 mg/ml BJOE, which was stored at 4°C for use.

Recombinant Human Interleukin-6 (rIL-6) protein (active) (Catalog Number: CSB-AP001741HU) was purchased from CUSABIO (CUSABIO TECHNOLOGY LLC, USA). The rIL-6 powder was briefly centrifuged prior to opening the vial to move the contents to the bottom. Then, it was dissolved in sterile distilled water to form a stock solution of 1 mg/ml, which was divided into working aliquots and stored at -20°C.

Commercial pills of LMV (GlaxoSmithKline, Middlesex, UK) were ground and dissolved in appropriate solutions as per the manufacturer’s instructions, and then filtered using 0.22-µm filters (Millipore Carrigtwohill, County Cork, Ireland). Based on the molecular weight and mass of LMV, the concentrations of the various stock solutions were estimated and diluted to the required concentrations.

Brusatol was purchased from rhawn-biotech (Shanghai, China; CAS No: 14907-98-3); bruceine B from weikeqi-biotech (Chengdu, China; CAS No: 25514-29-8); and bruceine A (CAS No: 25514-31-2), bruceantin (CAS No: 41451-75-6), and bruceantinol (CAS No: 53729-52-5) from Macklin-biotech (Shanghai, China). They were all dissolved using methanol into a concentration of 10 mg/ml.

### 2.3 Detection of HBV markers

Viral markers (HBsAg and HBeAg) in the cell supernatant were detected at the clinical laboratory of Shaoxing Maternity and Child Health Care Hospital using the Architect-i2000 system (Abbott Laboratories, USA) and HBs/eAg chemiluminescent microparticle immunoassay (CMIA) kits (Abbott Laboratories, Germany). HBV DNA in the cell supernatant or lysis solution was detected by real-time qPCR (LightCycler480 II, Roche) using a Taqman Probe-Fluorescence qPCR Kit (Sansure Biotech, China) (NMPA No: 20193400886). The quantification of biomarkers was conducted based on the criteria set by the manufacturer. The BJOE-treated group were analyzed and compared with the no BJOE treatment group (set as 1.0/100%).

### 2.4 Western-blot analysis

HepG2.2.15 cells were harvested at 72 h post BJOE treatment. Total proteins were extracted in lysis buffer (P0013B; Beyotime, Jiangsu, China) with inhibitors of proteases and phosphatases (P1260; Solarbio, Beijing, China) and quantified using a Bio-Rad protein assay kit (Bio-Rad). Cell lysates (35 mg/sample) were subjected to SDS-PAGE and transferred electrophoretically to a polyvinylidene difluoride membrane that was blocked with 5% nonfat milk in PBS with 0.1% Tween 20 (PBST). Followed by incubation with primary antibodies recognizing HBcAg (Abnova) or β-actin (Sigma-Aldrich) in appropriate dilutions. After three washes with PBST, peroxidase-conjugated secondary antibodies matched to the primary antibodies were added. Target proteins were visualized using ECL western-blot detection reagents (GE Healthcare, Little Chalfont, UK).

### 2.5 Cytometric bead array (CBA)

Cytokines consisting of interleukin-2 (IL-2), interleukin-4 (IL-4), interleukin-6 (IL-6), interleukin-10 (IL-10), tumor necrosis factor-α (TNF-α), and interferon-γ (IFN-γ) in culture medium supernatant were tested by flow cytometry (FCM) using CBA. According to the manufactures’ instructions, test specimens with 10-fold serial dilution standards and 1 negative control were prepared. Capture bead mixture buffer (25 μl/sample multiplied by the sample number) was centrifuged at 200 g for 5 min, and the supernatant was discarded. The harvested pellet was resuspended using an equal volume of beads buffer and incubated for 30 min. Then, 25 μl each of incubated beads, phycoerythrin-labeled anti-bead antibody, and samples were mixed and incubated in dark at room temperature for 2.5 h. The mixture was rinsed with 1 ml PBS buffer, followed by centrifugation at 200 g for 5 min. The pellet was resuspended with 100 μl PBS and then subjected to FACS. With reference to a standard curve, the concentration of each cytokine was determined. Each experiment was performed in triplicate and independently repeated 3 times.  

### 2.6 Transcriptome sequencing

Total RNA of HepG2 cells with or without 5 mg/ml BJOE treatment was isolated and purified using Trizol reagent (Invitrogen, Carlsbad, CA, USA). RNA concentration and integrity were assessed using NanoDrop 2000 (NanoDrop, Wilmington, DE, USA) and Agilent 2100, respectively. Transcriptome libraries were prepared using the AHTS Universal V8 RNA-seq Library Prep Kit for Illumina (Vazyme, China) according to the manufacturer’s instructions, and then sequenced on GenoLab M platform (GeneMind Biosciences Ltd., China) using a 150-cycle paired-end high-output sequencing mode.

### 2.7 Analysis of differentially expressed genes (DEGs)

Cutadapt (v1.18) was used to filter ribosomal RNA (rRNA) reads, adapter sequence, and ploy-N or low-quality reads (ratio of N greater than 5%; reads with more than 20% bases having quality Q value ≤ 20) to obtain clean reads. Then, the clean reads were mapped to the human reference genome sequence (GRCh38.p13) via HISAT2 (v2.2.1) software using default parameters. The alignment of files was conducted via samtools (v1.16) to remove reads with mapping quality (MAPQ) scores < 30. StringTie (v2.2.1) was used to perform expression quantification using default parameters. The differentially expressed genes (DEGs) with |log2(fold change)| ≥ 1 and with statistical significance (p.adj < 0.05) were selected using the R package DESeq2.

### 2.8 Protein–protein interaction (PPI) network analysis and hub gene identification

Search Tool for the Retrieval of Interacting Genes (STRING) (http://string-db.org/) database was used to explore the interaction among the upregulated DEGs following BJOE treatment. The threshold score for each PPI relationship pair was set to 0.4, indicating that an interaction with a combined score > 0.4 was considered statistically significant. Further, for hub genes identification, the gene degree was calculated using the degree algorithm of cytoHubba mode from cytoscape (v3.9.0) software. In this study, genes with the largest degree value were considered as hub genes in the PPI work.

### 2.9 Functional enrichment analysis

Gene Ontology (GO) enrichment analysis was conducted using the “clusterProfiler” R package. The results of enriched biological process (BP) were shown in a heatmap-like plot based on the gene sets with an adjusted p-value < 0.05.

### 2.10 Statistical analysis

Statistical analysis was performed using GraphPad Prism 5.01 (GraphPad Software, Inc., USA). The results of the three independent experiments are presented as mean ± standard deviation. Differences between two independent samples were determined using the two-tailed Student’s t test, and differences in multiple comparisons were determined using one-way analysis of variance (ANOVA) followed by the Least Significant Difference *post hoc* test. For all tests, *P<0.05, **P<0.01, and ***P<0.001 were considered to indicate a statistically significant difference.

## 3 Results

### 3.1 Effects of different concentrations of BJOE on cell viability of hepatic cell lines determined by CCK-8

**Figure 1:**
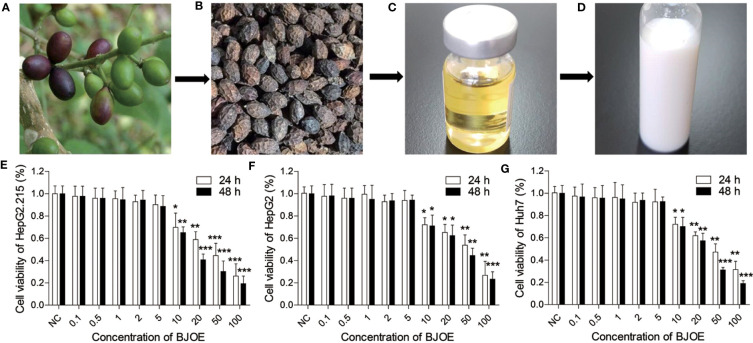
Flow chart of BJOE and the Effects of different concentrations of BJOE on cell viability. **(A)** Fresh BJ seeds. **(B)** Dried BJ seeds. **(C)** BJ oily liquid. **(D)** BJ oil emulsion. **(E)** The viability of HepG2.215 was detected following treatment with different BJOE concentrations for 24 h and 48* h*. **(F)** The viability of HepG2 was evaluated following treatment with different BJOE concentrations for 24 h and 48* h*. **(G)** The viability of Huh7 was detected following treatment with different BJOE concentrations for 24 h and 48* h*. *P<0.05, **P<0.01, and ***P<0.001 were considered to indicate a statistically significant difference.

As shown in **Figure 1A**, mature BJ seeds (the fruits of BJ plant) were harvested and gain dried (**Figure 1B**). Then, crude BJ oil was extracted from the mature dried BJ seeds using super critical fluid carbon dioxide, and after degumming, alkali refining, discoloration, and deodorization, refined BJ oil was obtained (**Figure 1C**). As introduced in the materials and methods sections, BJOE (**Figure 1D**) was produced by the emulsification of BJ oil with Tween-80, glycerinum, and sterilized distilled water.

The viability of HepG2.215, HepG2, Huh7, and LO2 (human normal liver cell) were detected following treatment with different BJOE concentrations for 24 h and 48* h*. CCK-8 assay revealed that BJOE inhibited the growth of HepG2.215 (**Figure 1E**), HepG2 (**Figure 1F**), and Huh7 (**Figure 1G**) in a concentration- and time-dependent manner at concentrations ≤ 5 mg/ml. The treatment with 5 mg/ml BJOE for 24 h and 48 h did not significantly affect the OD value of hepatic cell lines. These results indicated that > 5 mg/ml of BJOE was cytotoxic; thus, a BJOE concentration ≤ 5 mg/ml was selected for the antiviral experiments to avoid BJOE cytotoxicity.

### 3.2 BJOE inhibited HBV replication and antigen expression

**Figure 2:**
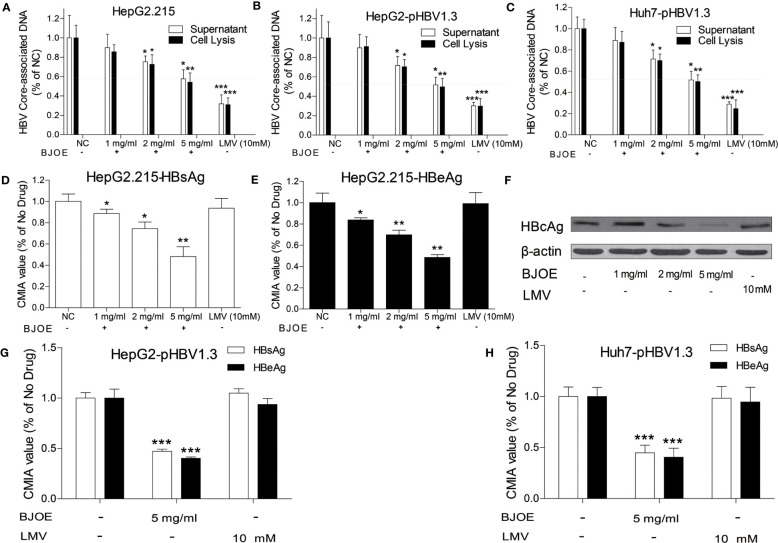
BJOE inhibited the replication and antigen expression of HBV. After treatment with different BJOE concentrations, HBV biomarkers were detected. **(A)** HBV DNA in the supernatant and cell lysis solution of HepG2.215 was evaluated by qRT-PCR. **(B)** HBV DNA in the supernatant and cell lysis solution of transfected HepG2 was evaluated by qRT-PCR. **(C)** HBV DNA in the supernatant and cell lysis of transfected Huh7 was calculated by qRT-PCR. **(D)** HBsAg in the supernatant of BJOE-treated HepG2.215 was analyzed by CMIA. **(E)** HBeAg in the supernatant of BJOE-treated HepG2.215 was analyzed by CMIA. **(F)** HBcAg in the cell lysis solution of BJOE-treated HepG2.215 was assessed by western blotting. Beta-actin was used as a loading control. One representative out of three experiments was shown. **(G)** HBs/eAg in the supernatant of pHBV1.3-transfected and BJOE-treated HepG2 was analyzed by CMIA. **(H)** HBs/eAg in the supernatant of pHBV1.3-transfected and BJOE-treated Huh7 was analyzed by CMIA. *P<0.05, **P<0.01, and ***P<0.001 were considered to indicate a statistically significant difference.

HepG2 and Huh7 were transfected with pBSK-HBV1.3-WT plasmid to establish HBV replication. Similarly, HepG2.215 could also produce various HBV particles, antigens, and forms of viral DNA as the transfected HepG2/Huh7. They were all treated with different doses of BJOE. Compared with the blank control and the LMV-treatment control, BJOE mildly and homogeneously reduced HBV replication in HepG2.215 (**Figure 2A**), HepG2 (**Figure 2B**), and Huh7 (**Figure 2C**) in a dose-dependent manner. It was estimated that approximately 40% of the HBV replication capacity was suppressed by a maximal dose of BJOE without cytotoxicity (5 mg/ml).

HBsAg and HBeAg in culture supernatant were analyzed with CMIA, and HBcAg was analyzed by western blot. As shown in **Figure 2**, BJOE significantly reduced HBsAg (**Figure 2D**) and HBeAg (**Figure 2E**) in the culture supernatant and HBcAg (**Figure 2F**) in cell lysis mixture of HepG2.215 in a dose-dependent manner, while LMV was not effective to all three types of antigens, consistent with previous reports that NAs are not effective for reducing HBs/e/cAg expression at the cell line level (Qin et al., 2013). A similar conclusion was reached after HBs/e antigen detection in HepG2 and Huh7, as BJOE strongly restrained HBs/eAg expression in the culture supernatant of HepG2 (**Figure 2G**) and Huh7 (**Figure 2H**).

### 3.3 BJOE regulated cytokine secretion in cell culture supernatant

**Figure 3:**
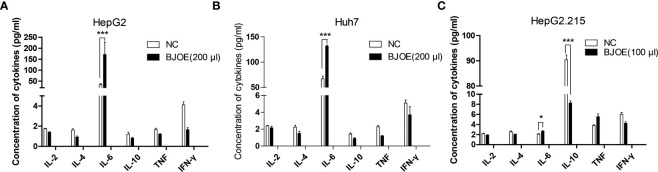
BJOE regulated cytokines secretion in the supernatant of different cultured cells. **(A)** IL-2, IL-4, IL-6, IL-10, TNF-α, and IFN-γ in the supernatant of BJOE-treated HepG2 were detected by FCM. **(B)** The level of cytokines in the supernatant of BJOE-treated Huh7. **(C)** The level of cytokines in the supernatant of BJOE-treated HepG2.215 were determined by CBA. ***P<0.001 was considered to indicate a statistically significant difference.

Cytokines in the supernatant of hepatoma cell lines (HepG2.215, HepG2, Huh7) were detected by FCM. In the absence of HBV replication, BJOE remarkably enhanced the secretion of IL-6 in HepG2 (**Figure 3A**) and Huh7 (**Figures 3A, B**), whereas five other cytokines were lowered in different degrees in HepG2 and Huh7. The reson why IL-6 only increased slightly after BJOE treatment in HepG2.215 is unclear. Furthermore, the background expression of IL-6 in HepG2.215 was much weaker than in other cell lines, while the expression of IL-10, which was much higher than others in HepG2.215, was significantly reduced by BJOE (**Figure 3C**).

### 3.4 IL-6 was stimulated by BJOE and inhibited HBV replication

**Figure 4:**
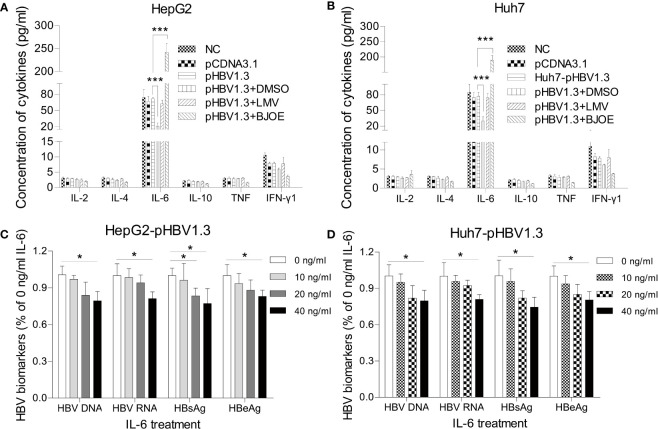
BJOE inhibited HBV replication through upregulation of IL-6. **(A)** IL-2, IL-4, IL-6, IL-10, TNF-α, and IFN-γ in the supernatant of transfected/untransfected HepG2 were assessed with CBA by FCM after treatment with DMSO, LMV, and BJOE. **(B)** Cytokines in the supernatant of transfected/untransfected Huh7 were detected with CBA after treatment with DMSO, LMV, and BJOE. **(C)** HepG2 transfected cells were left untreated or exposed to 10, 20, or 40 ng/ml of rIL-6. The HBV DNA and RNA were quantified using RT-qPCR. HBsAg and HBeAg in the supernatant were evaluated by CMIA. **(D)** Huh7 cells were treated with rIL-6, as described above. The cytoplasmic HBV DNA and RNA were extracted and quantified. The extracellular HBsAg and HBeAg were analyzed by CMIA. *P<0.05 and ***P<0.001 were considered to indicate a statistically significant difference.

To clarify the relationship between BJOE, IL-6, IL-10, and HBV, HepG2 and Huh7 transfected with pBSK-HBV1.3-WT were treated by LMV, BJOE, and DMSO. As shown in **Figure 4A**, transfection itself did not significantly influence the cytokines expression in HepG2. However, DMSO, which could enhance HBV replication, slightly downregulated IL-6 secretion, while BJOE with anti-HBV effects strongly upregulated IL-6 secretion, similar to the effects in Huh7 (**Figure 4B**). These findings suggest that BJOE inhibited HBV replication by upregulating IL-6.

To elucidate the actual mechanism, HepG2 and Huh7 transfected with pBSK-HBV1.3-WT were treated with four different concentrations (0, 10, 20, 40 ng/mL) of recombinant IL-6 (rIL-6). Then, cells and supernatants were harvested 72 h post-transfection and processed in parallel to extract HBV DNA and RNA. Exposure to exogenous rIL-6 moderately reduced the levels of HBV DNA, RNA, HBsAg, and HBeAg at 20 and 40 ng/ml in HepG2 (**Figure 4C**) and Huh7 (**Figure 4D**). IL-6 was previously reported to be an immunoregulatory cytokine in HBV infection, as it could inhibit HBV transcription and replication directly, which is consistent with our results.

**Figure 5:**
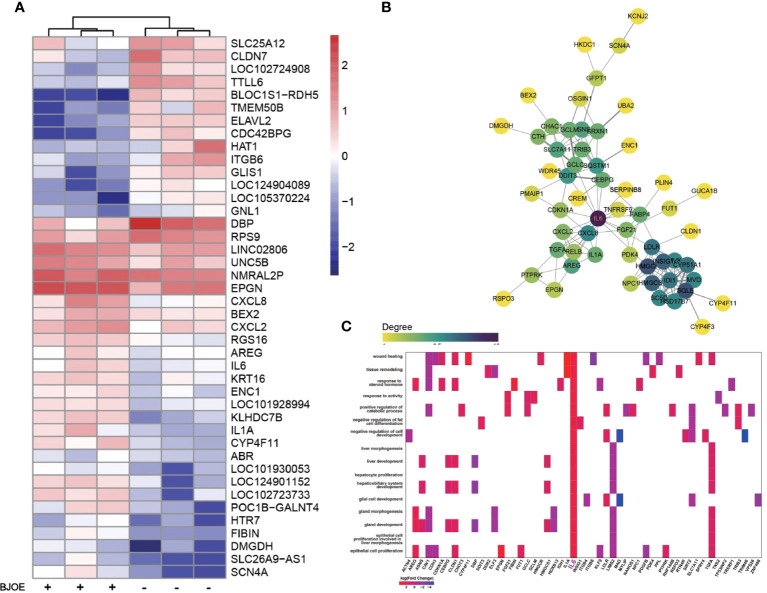
Upregulation of IL-6 was confirmed by transcriptome sequencing. **(A)** Heat map of DEGs induced by BJOE in HepG2 cells. Red and blue represent relative up -or downexpression of genes, respectively. **(B)** PPI network of hub genes in the upregulated DEGs. The darker the color, the more powerful the critical degree. **(C)** GO enrichment analysis based on the DEGs following BJOE treatment. The heatmap-like plot showed all enriched biological processes associated with IL6. Gradient color indicates the log2 (fold change) value of DEGs.

As shown in **Figure 5A**, IL-6 was upregulated by BJOE, which was consistent with the result of the cytokines test. To explore the interactions among the upregulated DEGs following BJOE treatment, STRING database was used. Genes with larger degree value in darker color were considered as hub genes in the PPI work. As shown in **Figure 5B**, IL-6 was the key gene, demonstrating an extremely active interaction with others. GO enrichment analysis was performed based on the DEGs (**Figure 5C**), and IL6 was found to be associated with many important biological processes, especially liver-related processes such as proliferation and morphogenesis of hepatocytes, hepatic function, and others.

### 3.5 BJOE was effective on HBV LMV-MT

**Figure 6:**
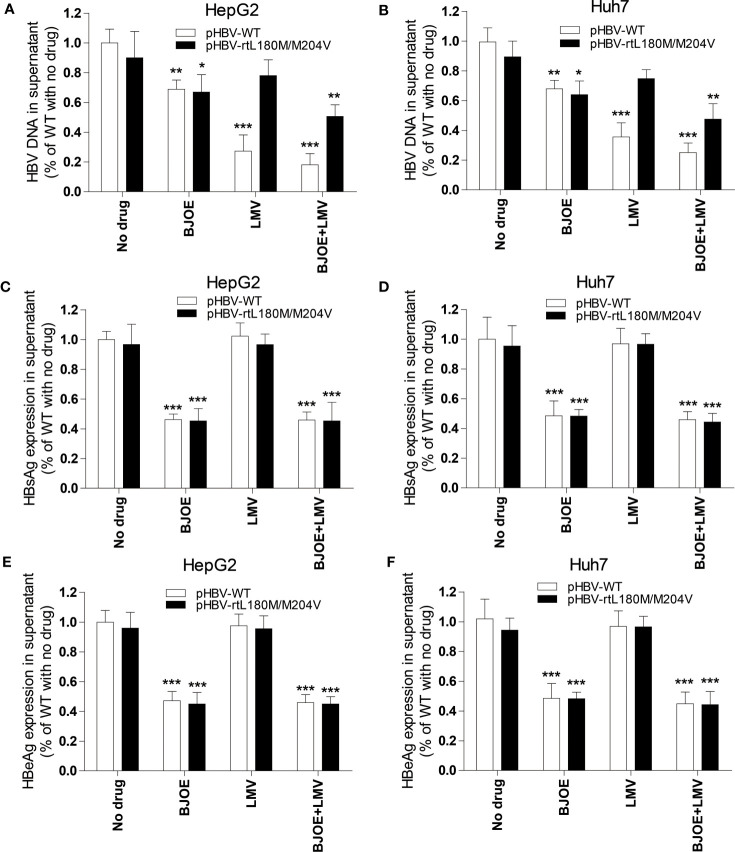
BJOE was effective on both WT and LMV-MT HBV. After transfection with pHBV1.3-WT or pHBV1.3-rtL180M/M204V and BJOE treatment, HBV biomarkers were analyzed. **(A)** HBV DNA in the supernatant of HepG2 was evaluated by qRT-PCR. **(B)** HBV DNA in the supernatant of Huh7 were evaluated by qRT-PCR. **(C)** The level of HBsAg production and secretion in the supernatant of HepG2 was evaluated by CMIA. **(D)** The expression of HBsAg in the supernatant of Huh7 was assessed by CMIA. **(E)** Secreted HBeAg in the supernatant of HepG2 was evaluated by CMIA. **(F)** HBeAg in the supernatant of Huh7 was detected by CMIA. *P<0.05, **P<0.01, and ***P<0.001 were considered to indicate a statistically significant difference.

To determine whether BJOE could control MT-HBV, pBSK-HBV1.3-WT with a replication-competent wild-type 1.3-fold over-length genotype A genome (GenBank accession No. U95551, ayw) was used as a backbone to construct pBSK-HBV1.3-rtL180M/M204V (rtM204V: a classic LMV resistance mutation; rtL180M: LMV adaptive mutation). After transfection of pBSK-HBV1.3-rtL180M/M204V or pBSK-HBV1.3-WT for the control, HepG2/Huh7 cells were treated with LMV, BJOE, or both, and HBV biomarkers in supernatants were assessed. As shown in **Figure 6**, 5 mg/ml BJOE inhibited the replication of both WT and LMV-MT, while LMV showed a selectively superior effect on WT but not on LMV-MT in HepG2 (**Figure 6A**) and Huh7 (**Figure 6B**). When used in combination, their anti-HBV effect was enhanced. Thus, 5 mg/ml BJOE could significantly inhibit HBsAg and HBeAg expression of either WT or LMV-MT in HepG2 (**Figures 5C**, **6E**) and Huh7 (**Figures 6D, F**), whereas LMV had no effect on HBs/eAg expression.

### 3.6 Bruceine B can induce IL-6 secretion and inhibit HBV

**Figure 7:**
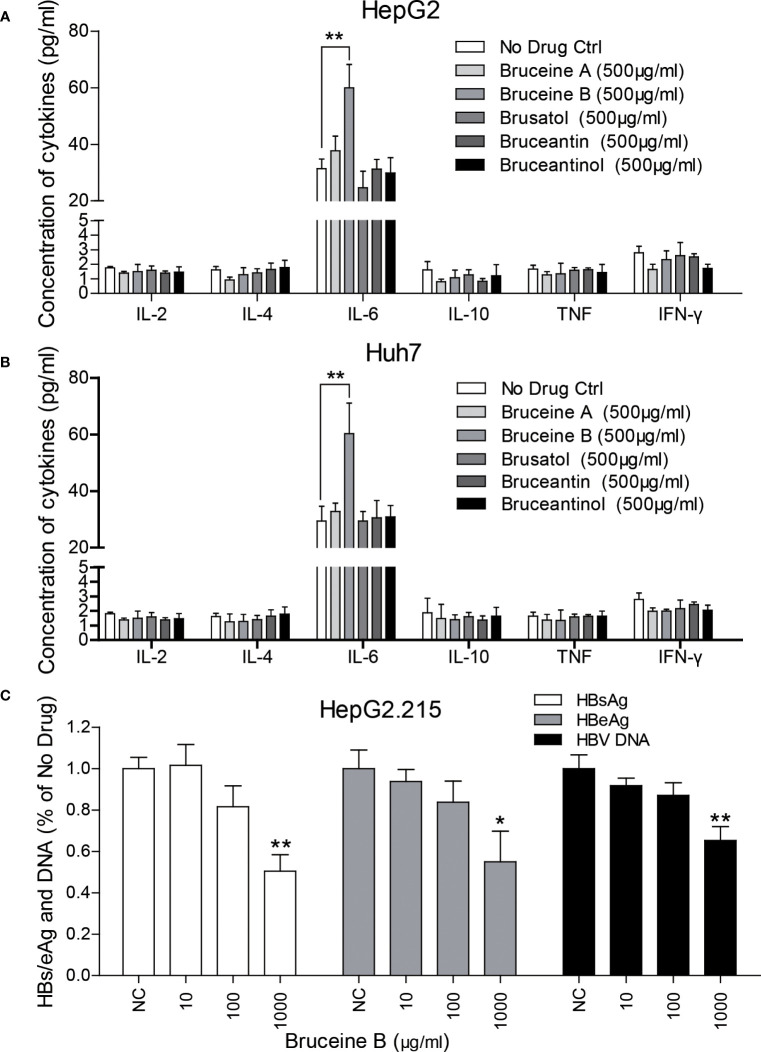
Identification of key components in BJOE responsible for IL-6 induction and the anti-HBV effect. **(A)** HepG2 **(B)** Huh7 were treated with 500 μg/ml brusatol, bruceine **(A)**, bruceine **(B)**, bruceantin, and bruceantinol, respectively. Cytokines in the supernatant of cultured medium were analyzed. **(C)** The anti-HBV effect of bruceine B were evaluated in HepG2.215. *P<0.05 and **P<0.01 were considered to indicate a statistically significant difference.

Cells were cultured, seeded into 6-well plates, and treated with different concentrations of brusatol, bruceine A, bruceine B, bruceantin, and bruceantinol, respectively, and a proportionate volume of methanol was set as the negative control (NC). Cytokine analysis of the supernatant showed that bruceine B significantly induced IL-6 compared to the others in HepG2 (**Figure 7A**) and Huh7 (**Figure 7B**). Then, their anti-HBV effect was evaluated in HepG2.215. As expected, bruceine B inhibited both HBs/eAg and HBV DNA in a dose-independent manner (**Figure 7C**), while the other four treatments had no effect (data not shown).

## 4 Discussion

CHB infection is associated with a high risk of severe liver disease and is one of the major causes of liver cancer and the related mortality (Qin et al., 2020). The interaction between HBV infection and the host defense system determines the pathogenesis or cure. Complete cure can be achieved by inhibition of HBV replication intermediates, complete decay of covalently closed circular DNA (cccDNA), and total blockade of re-infection. Generally, current therapeutic modalities aim to achieve functional cure, characterized by HBsAg loss, anti-HBs seroconversion, and cccDNA inactivation (Pei et al., 2017). Clinical treatment of HBV still depends on IFNs and NAs, but neither can eliminate cccDNA to achieve functional cure (Xia and Liang, 2019). Unfortunately, functional cure is still rarely achieved with the treatments available (Durantel and Zoulim, 2016).

BJOE was derived from BJ, a cheap and useful Chinese herbal medicine. Several compounds could be isolated from BJ, namely bruceine D, bruceine E, para-hydroxybenzoic acid, vanillic acid, luteolin, protocatechuic acid, and gallic acid (Ablat et al., 2017). Previous studies had shown that BJOE owned dramatic antiphytoviral activity. In this study, it was found that ≤ 5 mg/ml of BJOE significantly inhibited cell proliferation in a dose- and time-dependent manner; thus, this concentration was chosen as the maximal dose in the present study. Based on the cell model of HBV replication, BJOE significantly inhibited HBV DNA and HBs/e/cAg expression in a dose-dependent manner. Due to different antiviral mechanism, not only WT but also LMV-MT HBV were sensitive to BJOE, while NAs become ineffective as soon as resistance mutations develop.

To gain detailed insight into the anti-HBV effects of BJOE, cytokine levels in the supernatant of different cell lines were measured. It was shown that IL-6 was upregulated at varying degrees after BJOE treatment, unlike the other five cytokines that were downregulated at different levels in liver-originated. After IL-6 was found to be stimulated by BJOE, transcriptome sequencing was implemented to generate the map of genic differential expression of BJOE treatment, the upregulation of IL-6 was further confirmed (**Figure 5A**). In addition to IL-6, biogenesis of lysosomal organelles complex-1, subunit 1 (BLOC1S1) decreased the most. BLOC1S1, a common component of BLOC and BORC multiprotein complexes, modulates lysosome content and lipid handling independent of autophagy, indicating that lysosomal lipolysis is dependent on the cellular content of functional lysosomes (Wu et al., 2021). Sodium Voltage-Gated Channel Alpha Subunit 4 (SCN4A), Solute Carrier Family 26 Member 9 (SLC26A9), and Dimethylglycine Dehydrogenase (DMGDH) increased significantly after BJOE treatment. According to PPI network analysis and hub gene identification, as shown in **Figure 5B**, IL6 with the largest degree value was considered as the hub gene. Combined with GO enrichment analysis (**Figure 5C**), IL6 probably plays a key role in many biological processes.

Contrary to BJOE with anti-HBV effect, DMSO enhanced HBV replication mildly in accordance with previous study, accompanied by a rapid decrease in IL-6 secretion. The anti-HBV effect of BJOE was slightly stronger in HepG2/Huh7 than in HepG2.215 (**Figures 2A–C**), which might have resulted from the degree of IL-6 upregulation. IL-6 was reported to be a multifunctional α-helical cytokine that mediates cell growth and differentiation in various tissues and plays important roles in immune response, acute phase reactions, hematopoiesis, bone metabolism, and cancer progression. In fact, IL-6 reduced HBV infection without IFN-α/β and IFN-γ involvement (Kuo et al., 2009), and it can inhibit HBV pgRNA and subgenomic HBV RNAs transcription by reducing cccDNA-bound histone acetylation (Palumbo et al., 2015). By activating MAPK ERK and JNK, IL-6 downregulated the expression of HNF4α and HNF1α, which are the key transcription factors of HBV, followed by the inhibition of HBV transcription and replication (Hösel et al., 2009). IL-6 also inhibits HBV entry by downregulating NTCP, a cellular receptor of HBV (Bouezzedine et al., 2015). Taken together, our results provided important information to confirm that IL-6 may be an immunoregulatory cytokine in HBV infection. In line with previous research, rIL-6 showed certain curative anti-HBV effect in the present study. IL-10 was thought to be a key cytokine regulating the immune response to HBV infection. The function of exhausted HBV-specific CD8^+^ T-cells could be restored by reduction of IL-10 (Das et al., 2012; Liu et al., 2016). Interestingly, IL-10 inhibited other proinflammatory cytokines such as IFN-γ, TNF-α, and IL-6, and further affected the antiviral immune response (Walter, 2014). This may explain why the background expression of IL-6 in HepG2.215 with higher IL-10 expression level was much lower, and the stimulation effect by BJOE of IL-6 was not as obvious as in other cell lines. This might suggest that the anti-HBV effect of BJOE in HepG2 and Huh7 was mainly driven by increased IL-6, whereas the effect in HepG2.215 was driven by both increased IL-6 and decreased IL-10. BJOE is a complicated mixture, consisting of dozens of ingredients. Five main ingredients including brusatol, bruceine A, bruceine B, bruceantin, and bruceantinol were selected for further study. Interestingly, it was found that the effects of IL-6 induction and HBV inhibition were mainly caused by bruceine B.

Complete and specific immune responses can completely eradicate HBV in infected hepatocytes (Lan et al., 2015). Cytokines play an important role in the immune system and have been shown to control HBV at a post-transcriptional level, contributing to HBV cure in different models (Xia and Protzer, 2017). IL-6, stimulated by BJOE, participates in controlling HBV infection in many aspects. BJOE with non-toxic side effects and dual effects against WT/MT-HBV is an inexpensive commonly-used, pure natural plant extract and has already been used clinically as an anti-tumor agent. In addition to liver disease, HBV was also associated with many types of cancers, including non-Hodgkin’s lymphoma, pancreatic cancer, gastric cancer, nasopharyngeal carcinoma, lung cancer, esophageal cancer, ovarian cancer, and breast cancer (Li et al., 2020). It our recent study, NTCP, the receptor of HBV, was highly expressed in breast cancer which can be infected by HBV (Qin et al., 2020). As an antineoplastic, BJOE can also inhibit HBV and could be a better alternative for HBV-infected cancer patients. Further research is required to elucidate the detailed mechanisms of BJOE and bruceine B in the process of HBV infection and to verify its anti-HBV effect *in vivo*.

## Data availability statement

The data presented in the study are deposited in the China National GeneBank DataBase (CNGBdb) (https://db.cngb.org/) repository, accession number: CNP0004177.

## Author contributions

BQ provided fundamental support; BQ, LF and JD designed the research; BQ, SS, JL, WY, LF conducted the research experiments; BQ analyzed the data and wrote the paper.
